# Systemic racism: individuals and interactions, institutions and society

**DOI:** 10.1186/s41235-021-00349-3

**Published:** 2021-12-20

**Authors:** Mahzarin R. Banaji, Susan T. Fiske, Douglas S. Massey

**Affiliations:** 1 Harvard University, Cambridge, MA USA; 2 Princeton University, Princeton, NJ USA

## Abstract

Systemic racism is a scientifically tractable phenomenon, urgent for cognitive scientists to address. This tutorial reviews the built-in systems that undermine life opportunities and outcomes by racial category, with a focus on challenges to Black Americans. From American colonial history, explicit practices and policies reinforced disadvantage across all domains of life, beginning with slavery, and continuing with vastly subordinated status. Racially segregated housing creates racial isolation, with disproportionate costs to Black Americans’ opportunities, networks, education, wealth, health, and legal treatment. These institutional and societal systems build-in individual bias and racialized interactions, resulting in systemic racism. Unconscious inferences, empirically established from perceptions onward, demonstrate non-Black Americans’ inbuilt associations: pairing Black Americans with negative valences, criminal stereotypes, and low status, including *animal* rather than *human*. Implicit racial biases (improving only slightly over time) imbed within non-Black individuals’ systems of racialized beliefs, judgments, and affect that predict racialized behavior. Interracial interactions likewise convey disrespect and distrust. These systematic individual and interpersonal patterns continue partly due to non-Black people’s inexperience with Black Americans and reliance on societal caricatures. Despite systemic challenges, Black Americans are more diverse now than ever, due to resilience (many succeeding against the odds), immigration (producing varied backgrounds), and intermarriage (increasing the multiracial proportion of the population). Intergroup contact can foreground Black diversity, resisting systemic racism, but White advantages persist in all economic, political, and social domains. Cognitive science has an opportunity: to include in its study of the mind the distortions of reality about individual humans and their social groups.

## Introduction

### Significance

American racial biases persist over time and permeate (a) institutional structures, (b) societal structures, (c) individual mental structures, (d) everyday interaction patterns. Systemic racism operates with or without intention and with or without awareness. But because these responses are based on socially defined racial categories, they are racialized, and because they are negative, they reveal the roots of racism. At the level of most behavior, they are also controllable, even if many non-Black people rarely notice these relentless patterns. Systemic racism is a unified arrangement of racial differentiation and discrimination across generations. Understanding these formidable challenges is necessary to understand and then dismantle them. Cognitive science can illuminate the fine-grained levels of inbuilt racial bias because it has the methods and the theories to do so. Moreover, studying racial bias is interesting; it will improve the science; and it is the obvious path to ensuring a mutually respectful, peaceful society that flourishes economically, politically, and socially.

At the Editor’s invitation, this article presents the social and behavioral science of systemic racism to a cognitive science audience. The tutorial defines systemic racism, describes its origins in US history, shows how the resulting racialized societal structures have become built-in cognitive structures that propagate in social interactions, resisting change. But these very societal-cognitive-social features can also be agents for change.

### Definition

Systemic racism is said to occur when racially unequal opportunities and outcomes are inbuilt or intrinsic to the operation of a society’s structures. Simply put, systemic racism refers to the processes and outcomes of racial inequality and inequity in life opportunities and treatment. Systemic racism permeates a society’s (a) institutional structures (practices, policies, climate), (b) social structures (state/federal programs, laws, culture), (c) individual mental structures (e.g., learning, memory, attitudes, beliefs, values), and (d) everyday interaction patterns (norms, scripts, habits). Systemic racism not only operates at multiple levels, it can emerge with or without animus or intention to harm and with or without awareness of its existence. Its power derives from its being integrated into a unified system of racial differentiation and discrimination that creates, governs, and adjudicates opportunities and outcomes across generations. Racism represents the biases of the powerful (Jones, 1971), as the biases of the powerless have little consequence (Fiske, 1993).^1^

### Preview

We highlight the “inbuilt” aspect of systemic racism to be its signature feature and the touchstone necessary to understand the nature of systemic racism and its resistance to awareness and change. We begin with the concept’s more traditional domains: institutional and societal systems. Then, given the current venue, we expand the levels of analysis to include individual mental systems that have built in those systems of inequalities. We close with the interaction of those minds in social behavior, which can either maintain or change racial systems.

*Institutions and Society*. As the first section explains, the term *systemic racism* has traditionally referred to systems that uphold racism via institutional power (Feagin, 2006), with stark examples of what is also called *institutional racism* (Jones, 1972) visible in inequities in housing and lending, as well as more broadly in access to finance, education, healthcare, and justice. This section focuses on the institutional level in depth, as it provides the strongest evidence of systemic racism. At an even more macro s*ocietal* level, however, the inbuilt aspect of systemic racism is evident in race-based demarcations created by large-scale state and federal programs, which offer levers either to increase or decrease systemic racism. To remain within the scope of the paper, we consider the structures of *institutional* and *societal* racism in a single section.

*Individuals and Interactions*. In tandem with the previous section, this section focuses on individual bias and interactional racism, together bringing into view the inbuilt nature of systemic racism. To expand on this inclusive view of systemic racism, we end by reviewing what we know about the individual human being, alone and interacting with others. *Individuals* are agentic entities, the primary actors within all systems of life and living. Their attitudes (preferences, prejudices), beliefs (stereotypes), and behaviors (discrimination) are inbuilt or intrinsically enmeshed into the foundation of the mental systems that feed systemic racism. At the individual level, “inbuilt” refers to the common psychological processes that represent race in the minds of individuals. This evidence reveals systemic race bias.

Note that, here, we use slightly different terms: Systemic Racism refers to much of the sociological, demographic, and historic material as well as anything in the psychological section that is explicit and conscious racism. Systemic Race Bias is about implicit cognition—people who may not be aware of the harm they may cause. Implicit race bias does not mean a person is a racist. In this view, keeping racism and bias separate as terms seems advisable. Others view even unexamined racism as systemic racism in its individual manifestation. Each section elaborates on the meaning of racism in that context.

Individual racial bias propagates through both face-to-face and virtual interactions within families, classrooms, playfields, and workplaces, both verbally and non-verbally. Individual minds create and consume racial representations in books, social media, and entertainment.^2^ We focus here on everyday interactions that convey disrespect and distrust of Black Americans.

### Why? Role for psychological science in studying systemic racism

Individual humans are the creators and consumers of thoughts, feelings, and behaviors, but also the policies and practices that lie at the heart of systemic racism. Psychology as a field has historically remained silent on the topic of systemic racism, per se (e.g., Guthrie, 2004, “Even the rat was white”; for exceptions, see: Jones, 1971; DuBois, 1925). Perhaps psychologists have regarded systemic racism to be a form of institutional racism and hence in the bailiwick of social scientists who study institutions and society, not individuals. Nonetheless, we attempt here to include individual minds and face-to-face interaction as playing a role. This goal has precedents: Early scholars who straddled disciplines, such as George Herbert Mead (1934, p. 174), would likely find our attempt to be quite compatible with his stance that mind and society must be considered in intertwined fashion.

Today, psychologists are increasingly attempting to bridge the divide between the individual mind and society. Cultural psychology, for example, has attempted to analyze racism as the “budding product of psychological subjectivity and the structural foundation for dynamic reproduction of racist action” (Salter, Adams & Perez, 2018, p. 151). This dynamic can emerge in individual racist actions (with or without awareness) that are fitted into the structure of everyday life and perpetuate systemic racism. Interpersonal interactions bridge individual and collective representations of race. Individual minds, sharing some notions about each other’s salient identities (e.g., probable race, gender, age) treat each other according to social norms, cultural habits, and cultural scripts. In the case of race, these individual mental representations and social interaction patterns rarely benefit Black participants facing Whites.

### “Inbuilt”: A useful metaphor guiding the essay

There are these two fish swimming along and they happen to meet an older fish swimming the other way, who nods at them and says ’Morning, boys. How’s the water?’ And the two young fish swim on for a bit, and then eventually one of them looks over at the other and goes ‘What the hell is water?’

Wallace, 2009

The fable highlights a simple idea—that the most fundamental feature of any system may be so completely pervasive that it ceases to be perceptible or when perceptible, fails to be recognized in its true form. This paradox creates a challenge for social and behavioral scientists, who must not only generate evidence about the complexities of systemic racism, but we must also confront unthinking rejection of that evidence. Other scientists face similar challenges in documenting their own complex phenomena, such as the resistance faced by the theory of evolution or the denial of evidence about climate change.

In most cases, evidence eventually reaches a tipping point, after which it ceases to be denied and even becomes sufficiently commonplace that its previous denial itself is puzzling. An easy example is the denial of scientific evidence about the position of the earth in the solar system and its shape, with few arguments today (but not zero!) about a flat earth. However, we are far from that tipping point of knowledge and acceptance when it comes to the idea of systemic racism. This paper, then, is yet another attempt, by connecting across the individual, interactional, and institutional/societal levels, to shed light on its existence.

The obvious allegorical lesson from the fable about the fish is of course the ease of being ignorant of that which is pervasive. However, the fable also points out that not all the fish are ignorant of their surroundings. The older fish, swimming the same ocean as the young fish, seems to have figured out the truth about the substance that suffuses its environment so fully that it is imperceptible to its peers. Ignorance then, need not be the only guaranteed outcome, even when perception and awareness are hard. Hence, one section uses the term “unexamined” to describe controllable attention to or willful neglect of one’s own biases (see also Fiske, 1998). Social scientists commenting on resistance to socioeconomic inequality have used the term “clueless” (Williams, 2019), which is admittedly harsh but suggests that learning some facts would permit more evidence-based understanding. Regardless, the evidence for systemic racism, at the level of institutions and society or at the level of individuals and interactions, requires re-examining the taken-for-granted, whether the water we swim or the air we breathe.

## Systemic racism: the role of institutional and societal structures

Contemporary societal racism rests on Black–White segregation, historical and current. This first substantive section presents evidence that systemic racism has long pervaded US institutional and societal systems—creating a context for the minds of individuals within these systems, enabling an omnipresent neglect. First, this section shows that continued housing segregation by race obstructs Black opportunity and mobility, perpetuating racial disparities, challenging many Black Americans in ways White Americans never experience (Massey, 2020). At a societal level, Black disadvantage and White advantage come in part from residential hypersegregation (Massey & Tannen, 2015). More than any other racial group, Whites live in racially isolated neighborhoods (Rugh & Massey, 2014); and in the US neighborhood segregation translates directly into school segregation (Massey & Tannen, 2016; Owens, 2020). Both segregation and local funding undermine the quality of predominantly Black schools.

To elaborate these points, this section describes the historical context for US racism, territory likely to be less familiar to cognitive scientists. Our takeaway: Systemic racism pervades US social institutions, policies, and practices; later sections show how the societal structures make into the minds of the humans within these systems.

### History: segregation and systemic racism

To explain systemic racism, we start with the historical origins of race in the US—that is, the social/political/economic mechanisms that have maintained it over time. Race is baked into the history of the US going back to colonial times (Higginbotham, 1998; Jones, 1972, 1997) and continuing through early independence when slavery was quietly written into the nation’s Constitution (Waldstreicher, 2009). Although the 13th, 14th, and 15th Amendments to the Constitution ended slavery and granted due process, equal protection, and voting rights to the formerly enslaved, efforts to combat systemic racism in the US faltered when Reconstruction collapsed in the disputed election of 1876, which triggered the withdrawal of federal troops from the South (Foner, 1990).

The absence of federal troops to enforce Black civil rights enabled states in the former Confederacy to construct a new system of racial subordination known as Jim Crow (Packard, 2003). It rested on a simple principle: in any social encounter, the lowest status White person was superior to the highest status Black person. By law and custom, Black voting rights were suppressed, and Black Americans were socially segregated from Whites, relegated to menial occupations, inferior schools, dilapidated housing, and deficient facilities throughout Southern society. Any challenges to the Jim Crow system, perceived or real, were met with violence, often lethal, both within and outside the legal system (Tolnay & Beck, 1995).

From 1876 to 1900, 90% of all African Americans lived in the South and were subject to the dictates of the repressive Jim Crow system; 83% lived in poor rural areas, occupying ramshackle dwellings clustered in small settlements in or near the plantations where they worked. Although conditions were somewhat better for the 10% of African Americans who lived outside the South (68% in cities), anti-Black prejudice was widespread, racial discrimination was common and, as in the South, the prospect of racial violence was never far away (Sugrue, 2008).

Before, 1900, few African Americans lived in cities, and levels of urban racial residential segregation were modest. Black workers and servants generally lived within walking distance of their workplaces, and social contact between the races was common (Massey & Denton, 1993). At that time, the share of Blacks among city residents was small, and they were not perceived to be a threat to White hegemony, obviating the need for spatial segregation. The Great Black Migration of the twentieth century changed this status quo and transformed race relations in the US, making race truly a national rather than regional issue (Lemann, 1991). This transformation also created a new system of racial subordination based on Black residential segregation.

Between 1900 and 1970, millions of African Americans left the rural South in search of better lives in industrializing cities throughout the nation. As a result of this migration, by 1970 nearly half of all African Americans had come to live outside the South, 90% in urban areas (Farley & Allen, 1987). It was during this period of Black urbanization that the ghetto emerged as a structural feature of American urbanism, making Black residential segregation into the linchpin of a new system of racial stratification that prevailed throughout the US irrespective of region (Pettigrew, 1979).

Black out-migration from the South began slowly at first, but accelerated after 1914, when the onset of the First World War curtailed the arrival of workers from Europe. It accelerated again after 1917, when the US entered the war, boosting labor demand as conscription drew workers out of the labor force. The imposition of strict immigration restrictions in 1921 and 1924 guaranteed that Black workers and their families would continue to pour into cities during the economic boom of the 1920s (Wilkerson, 2010). The entry of ever-larger cohorts of impoverished Black laborers and sharecroppers into the nation’s cities unnerved White urbanites, prompting them to organize collectively by creating “neighborhood improvement associations.” These organizations pressured landlords not to rent to Black tenants and tried to convince Black home seekers that it was in their best interest to locate elsewhere, using persuasion and payoffs when possible but resorting to violence when these blandishments failed (Massey & Denton, 1993).

As the number of incoming Black migrants continued to rise despite these efforts, White city residents demanded that politicians act to “do something” about the perceived “Black invasion.” Officials in smaller towns and cities responded by enacting “sundown laws” that required all Blacks to leave town by sunset (Loewen, 2018). In large cities, legislators passed municipal ordinances that confined Black residents to a specific set of already disadvantaged neighborhoods and excluded them from all others. These ordinances were the functional equivalent of South Africa’s Group Areas Act, which underlay the establishment of that country’s apartheid system in, 1948. These ordinances were widely copied and were spreading rapidly from city to city when, in 1917, the Supreme Court declared them to be unconstitutional (Massey & Denton, 1993). Sundown laws, however, were never challenged in court and remained in force well into the Civil Rights Era.

The end of legally mandated neighborhood segregation in cities occurred just as Black migration surged in the aftermath of America’s entry into the First World War. The sudden influx of workers caused existing areas of Black settlement to fill up rapidly and eventually overflow into adjacent White areas, where the arrivals met with increasingly violent resistance. The violence peaked in the late teens as anti-Black race riots swept through the nation’s cities, culminating in the Great Chicago Race Riot of 1919 (Tuttle, 1970). Even established Black neighborhoods were not safe, as evidenced by the Tulsa Massacre of 1921, in which the prosperous Black neighborhood of Greenwood was systematically attacked and razed by mobs of White vigilantes, leaving thousands homeless and dozens, perhaps hundreds, killed (Madigan, 2001).

Shocked by the wanton destruction of property, the real estate industry moved to institutionalize racial discrimination in housing markets and assert control over the process of racial change in cities (Massey & Denton, 1993). In 1924, the National Association of Real Estate Brokers adopted a code of ethics stating that “a Realtor should never be instrumental in introducing into a neighborhood a character of property or occupancy, members of any race or nationality, or any individuals whose presence will clearly be detrimental to property values in that neighborhood” (Helper, 1969, p. 201). In 1927, the Chicago Real Estate Board devised a model racial covenant to block the entry of Blacks into White neighborhoods and offered it to other cities for adoption throughout the country (Massey & Denton, 1993). A racial covenant is a private contract in which property owners within a defined geographic area collectively agree not to rent or sell to African Americans. Once approved by a majority of property owners, the contract became enforceable, and violators could be sued in civil court.

As the real estate industry gradually assumed control of racial change in urban areas, racial violence abated and neighborhood transitions from White to Black came to be managed professionally by realtors who sought to minimize confrontation and maximize profits. As Black migration continued throughout the 1920s, recognized Black neighborhoods steadily increased in density as housing units were divided and subdivided. Basements, garages, attics, and even closets were converted into rental units. Eventually, however, no more living space could be squeezed into the confines of the existing ghetto. Realtors then conspired to move the residential color line, selecting an adjacent neighborhood for racial transition and initiating an institutionalized process known as “block busting” (Philpott, 1978).

Realtors began the process by choosing a few poor Black families just arrived from the rural South and obviously unused to city ways to be placed strategically into selected units within the targeted neighborhood. Agents then moved through the neighborhood block by block warning residents of a pending Black “invasion.” Panic selling ensued, enabling realtors to purchase homes cheaply for subdivision into smaller apartments, which were then leased at inflated rents to African Americans desperate for living space. Owing to these institutionalized practices, Black segregation levels steadily climbed through the 1920s and ghetto areas gradually expanded their boundaries through the profitable management of neighborhood racial turnover by realtors (Massey & Denton, 1993).

The exclusively private auspices of Black residential segregation ended with the onset of the Great Depression in 1929. When Franklin Roosevelt came to power with his New Deal in 1933, the nation was in the midst of a catastrophic banking crisis. Millions of middle-class homeowners had lost jobs and were in danger of defaulting on their mortgages, putting both their homes and their bankers at financial risk. In response, the Roosevelt Administration created the Home Owners Loan Corporation (HOLC) to help middle class homeowners refinance their mortgages using long-term, federally insured, low-interest loans (Jackson, 1985). Together the federal guarantees and extended amortization periods reduced monthly mortgage payments to affordable levels, saving both the banks and the homeowners from financial losses through foreclosure.

To qualify for the federal guarantees, however, HOLC loans had to meet certain government-mandated criteria. In addition to low interest rates, minimal down payments, and long amortization periods, lenders were obliged to consider the riskiness of the neighborhoods in which properties were located. To this end, HOLC officials worked with local realtors and bankers to create a series of Residential Security Maps for use in cities throughout the nation. These maps color-coded neighborhoods according to their creditworthiness. Green indicated a safe investment, yellow indicated caution, and red indicated excessive risk and hence ineligibility for HOLC lending. Black neighborhoods were invariably coded red, along with adjacent neighborhoods perceived to be at risk of Black settlement (Rothstein, 2017).

The HOLC lending program only helped the minority of families that already owned homes, however, and in order to spread housing wealth to a wider population and create jobs in the real estate and construction industries, in 1934 the Roosevelt Administration created a much larger loan program under the Federal Housing Authority. The FHA offered long-term loans to prospective home *buyers*, not just owners. As before, federally guaranteed loans had to meet federally mandated criteria, which evinced a strong anti-urban bias. Specifically, they excluded from eligibility all multiunit buildings, attached dwellings, row houses, and structures containing a business. These provisions effectively restricted FHA loans to single family houses on large lots, thus channeling housing investment away from central cities toward vacant land on the urban fringes (Jackson, 1985).

Reflecting the prejudices of the realtors, bankers, and builders who helped to design the program, FHA underwriters were also required to make use of the HOLC’s Residential Security Maps, formally institutionalizing the practice of redlining in real estate and banking and systematically cutting off investment in Black neighborhoods for decades to come. The FHA Underwriter’s Manual explicitly stated that “if a neighborhood is to retain stability, it is necessary that properties shall continue to be occupied by the same social and racial classes.” In addition to requiring the use of Residential Security Maps, the manual went on to advocate the use of racial covenants to protect FHA-insured properties. When a parallel loan program was created in the Veterans Administration by the 1944 Servicemen’s Readjustment Act, it adopted the FHA’s racialized practices and procedures (Katznelson, 2006).

The anti-urban biases and discriminatory practices built into federal loan programs had little effect on housing patterns during the 1930s and 1940s owing to the tiny amount of new residential construction that occurred during the Great Depression and Second World War. In the postwar period, however, FHA and VA lending drove forward a massive wave of suburban home construction that made new homes widely accessible to White but not Black households. Given high rents and home prices in central cities owing to the influx of workers during the war years, in the late 1940s and early 1950s it was cheaper to buy a brand-new house in the suburbs than to rent an apartment in the city (Massey & Denton, 1993).

The end result was a government-subsidized mass exodus of middle and working class White families from central cities to suburbs, creating a distinctly American urban configuration of Black cities surrounded by White suburbs. The homes left behind by the departing Whites seeking their piece of the American Dream in the suburbs were quickly occupied by Black in-movers coming to the city to take jobs in the still-vibrant urban manufacturing sector. Neighborhood turnover accelerated, and the nation’s urban Black ghettos rapidly expanded, both demographically and geographically (Massey & Denton, 1993).

Although neighborhood transitions in the 1950s and 1960s improved Black access to housing in the short term, in the long term the neighborhoods turned into poverty traps. Because of redlining and racial discrimination built into housing and credit markets by federal policies and private practices, once a neighborhood became Black, it was cut off from investment, ensuring that its housing stock and business infrastructure would progressively deteriorate. It also left the Black middle class without a means to finance the purchase of homes, and predatory lenders stepped into the resulting void.

Drawing on their own capital, these lenders purchased homes and then offered to “sell” them to middle class Black families by means of Loan Installment Contracts (Satter, 2009). LICs were essentially rent-to-own schemes with high interest rates, bloated monthly payments, and no property rights or transfer of title until the final contract payment was made. Any missed payment could bring about immediate eviction by the property owner, no matter how long the aspiring family had been making payments under the contract.

Other predatory investors also purchased ghetto properties to become landlords, subdividing them into ever-smaller units and leasing them to poor and working class Black tenants at inflated rents (Massey & Denton, 1993). Whether city housing was being sold under an installment contract or rented on usurious terms, however, the absentee owners could not themselves get loans to offset depreciation or purchase insurance policies to protect their properties, creating a strong financial incentive for landlords to defer maintenance, minimize capital investment, and extract high rents as long as possible until the properties deteriorated to the point of becoming uninhabitable.

As Black ghettos expanded geographically during the 1950s and 1960s in cities such as New York, Chicago, Philadelphia, Detroit, Cleveland, and St. Louis, they ultimately came to encroach on zones in which White elites had place-bound investments in universities, hospitals, museums, and business districts. In desperation, local politicians and civic leaders turned to state and federal agencies for help. Drawing on funding from the National Housing Act, they created locally controlled Urban Renewal Authorities with the power of eminent domain, thereby enabling White interests to gain control of the Black neighborhoods threatening their place-bound investments (Bauman, 1987; Hirsch, 1983). Once in control of the land, they evicted the residents, razed their homes, and demolished neighborhood businesses, replacing them either with large-scale middle-class housing projects or institutional developments that strategically blocked the expansion of the ghetto toward the threatened White properties, prompting James Baldwin to quip that “urban renewal means Negro removal” (Dickinson, 1963).

Because of a “one-for-one rule” embedded within the National Housing Act, for every unit of housing torn down in the name of renewal, planners had to identify another unit into which the displaced tenants could theoretically move. To satisfy this rule, local elites once again turned to the federal government, garnering additional funds authorized by the National Housing Act to construct large public housing projects for families displaced by renewal. Given that the displaced families were Black, it was politically impossible to build the housing project in a White district, so another Black neighborhood was targeted for renewal and torn down to build dense collections of high-rise projects that now had to house two neighborhood’s worth of displaced families (Massey & Denton, 1993).

This pairing of urban renewal and public housing did not itself increase the level of Black residential segregation (Bickford & Massey, 1991). Segregation levels were already high in the cities where this pairing occurred; but it did dramatically increase the spatial concentration of poverty within the ghetto by replacing relatively class-diverse Black neighborhoods and business districts with tightly packed blocks of high-rise projects in which being poor was a criterion for entry, yielding neighborhood poverty rates of 90% or more (Massey & Kanaiaupuni, 1993).

By 1970, high levels of Black residential segregation were universal throughout metropolitan America (Massey & Denton, 1993).^3^ As of 1970, 61% of Black Americans living in US metropolitan areas lived under a regime of hypersegregation (Massey & Tannen, 2015), a circumstance unique to Americans. Although in theory, segregation should have withered away after the Civil Rights Era, it has not. In 2010, the average index of Black–White segregation remained high and a *third of all Black metropolitan residents continued to live in hypersegregated areas* (Massey & Tannen, 2015). This reality prevails despite the outlawing of racial discrimination in housing (the 1968 Fair Housing Act) and lending (the 1974 Equal Credit Opportunity Act and the 1977 Community Reinvestment Act).

### Why does modern segregation persist, despite Whites’ reported racial attitudes improving?

Accompanying these legislative changes was a pronounced shift in White racial attitudes. In the early 1960s, more than 60% of White Americans agreed that Whites have a right to keep Blacks out of their neighborhoods. By the 1980s, however, the percentage had dropped to 13% (Schuman et al., 1998). The fact that discrimination is illegal, and White support for segregation has plummeted, begs the question of why segregation persists. The reasons are multiple.

First, although the Fair Housing Act banned discrimination in the rental and sale of housing, enforcement mechanisms in the original legislation were eliminated as part of a compromise to secure the bill’s passage (Metcalf, 1988). Federal authorities were likewise granted only limited powers to enforce the Equal Credit Opportunity Act and the Community Reinvestment Act (Massey & Denton, 1993).

Although overt discrimination in housing and lending has clearly declined in response to legislation, covert discrimination continues. Rental and sales agents today are less likely to respond to emails from people with stereotypically Black names (Carpusor & Loges, 2006; Hanson & Hawley, 2011) or to reply to phone messages left by speakers who “sound Black” (Massey & Fischer, 2004; Massey & Lundy, 2001). A recent meta-analysis of 16 experimental housing audit studies and 19 lending analyses conducted since 1970 revealed that sharp racial differentials in the number of units recommended by realtors and inspected by clients have persisted and that racial gaps in loan denial rates and borrowing cost have barely changed in 40 years (Quillian, Lee, & Honoré, 2020).

Audit studies, conducted across the social and behavioral sciences, include a subset of resume studies in which researchers send the same resume out to apply for jobs, but change just one item: the candidate’s name is Lisa Smith or Lakisha Smith. Then, they wait to see who gets the callback. The bias is clear: employers avoid “Black-sounding” names (Bertrand & Mullainathan, 2004). In fact, in both Milwaukee’s and New York City’s low-wage job market, Black applicants with no criminal background were called back with the same frequency or less as White applicants just released from prison (Pager, 2003; Pager, Western & Bonikowski, 2009).

That is, in the minds of hiring managers whose mental make-up is expected to be no different than the readers of this article, a White felon is equivalent to a Black non-felon. The same housing application, the same bank loan application, the same health data, the same behavior, lead to different outcomes depending on the race of the applicant, even though the decision-makers believe they are paying attention to the merits of the case and explicitly not to race, which most decision makers in these studies regard to be irrelevant to the decision.

What makes the problem of systemic racism so perverse is that “good people” with no explicit expression of we would call “racism” are the contributors to such decisions that produce widespread and unnoticed bias, resulting in systemic racism (Banaji & Greenwald, 2013). Racial discrimination continues because, although White support for Black segregation may have declined in principle, Whites nonetheless continue to harbor negative racial stereotypes about Black people*,* which limit their tolerance for integration in practice. Indeed, the willingness of Whites to enter or remain in a neighborhood declines steadily as the percentage of Black neighbors rises (Charles, 2003; Emerson, Chai & Yancey, 2001). And negative racial stereotyping of Black Americans strongly predicts White opposition to government efforts to enforce Black civil rights (Bobo, Charles, Krysan & Simmons, 2012).

In White American social cognition, as later sections elaborate, racial biases remain entrenched both explicitly (Moberg, Krysan & Christianson, 2019) and implicitly (Eberhardt, 2019). This extends to preferred neighborhoods*:* Residential searches are inevitably embedded within racialized expectations about neighborhoods and homes that reflect the racially segregated world that most Americans inhabit (Krysan & Crowder, 2017). The “correlated characteristics heuristic” relies on a single salient neighborhood trait—in this case racial composition—to represent an area’s acceptability. In White social cognition, the mere presence of Blacks denotes lower property values, higher crime rates, and struggling schools, irrespective of what the objective neighborhood conditions are (Krysan, Couper, Farley & Forman, 2009; Quillian & Pager, 2001, 2010). Although Whites in surveys and interviews say they welcome the presence of Black neighbors, in practice Whites avoid neighborhoods containing more than a few Blacks and confine their searches to overwhelmingly White residential areas exhibiting White percentages well above those they report in describing their “ideal” neighborhood on surveys (Krysan & Crowder, 2017).

Although rarely admitted, explicit prejudice against Black Americans has hardly disappeared. Google search frequencies on the epithet “nigger” for different metropolitan areas strongly predicted an area’s level of Black residential segregation (Rugh & Massey, 2014). This index of explicit racism also strongly predicts the degree to which a city’s suburbs are covered by restrictive density zoning regimes (Massey and Rugh (2018), a key proximate *cause* of both racial and class segregation (Rothwell & Massey, 2009, 2010). Owing to the persistence of discrimination, Black Americans are far less able that other Americans to translate their income attainments into residential mobility, greatly compromising their ability to access more integrated and favored neighborhoods (Massey & Denton, 1985). As of 2010, the most affluent Black Americans were still more segregated from Whites than the poorest Hispanics (Intrator, Tannen & Massey, 2016).

No other group in the history of the US has ever experienced such intense residential segregation in so many areas and over such a long period of time (Massey & Denton, 1993; Rugh & Massey, 2014). Systemic racism in federal housing policies (Katznelson, 2006), real estate (Helper, 1969), banking (Ross & Yinger, 2002), and insurance (Orren, 1974) has ensured a vicious cycle of racial turnover and neighborhood deterioration for most of the past century. As a result, many Black Americans have been compelled to live in societally isolated, economically disadvantaged, physically deteriorated neighborhoods produced and sustained by powerful external forces beyond their ability to control, the precise embodiment of systemic racism.

Because of racial residential segregation and the blocked mobility and spatial concentration of poverty it produces, neighborhoods have become the key nexus for the transmission of Black socioeconomic disadvantage over the life course and across the generations (Sharkey, 2013). Half of all Black Americans have lived in the poorest quartile of urban neighborhoods for two consecutive generations, compared with just 7% of Whites, a gap that cannot be explained by individual or family characteristics.

Whereas in the 1960s Black poverty was transmitted across generations by the inheritance of race and the discrimination and exclusion that came with it (Duncan, 1969), in the twenty-first century Black poverty is transmitted by the inheritance of place and the concentrated poverty it entails (Massey, 2013; Massey & Brodmann, 2014; Peterson & Krivo, 2010; Sampson, 2012; Sharkey, 2013). Black disadvantage with respect to income and social mobility is explained almost entirely by the poor neighborhood circumstances they experience (Chetty, Hendren, Jones & Porter, 2020; Massey & Brodmann, 2014). Racial residential segregation has become linchpin for systemic racism in the US in the twenty-first century (Massey, 2016, 2020).

Discussions of segregation typically highlight how it operates to increase the social isolation of Blacks, but in fact it does more to isolate Whites, who are by far the most spatially isolated group in the US. In 2010, the average Black metropolitan resident lived in a neighborhood that was 45% Black, but the average White metropolitan resident occupied a neighborhood that was 74% White (Massey, 2018), and in suburbs the figure rose to 80% (Massey & Tannen, 2017). As a result, the advantages of segregation to Whites and the disadvantages to Blacks are invisible to most White Americans.

Feagin (1999, p. 79), put this paradox into perspective by relating the experience of a British immigrant’s confrontation with the realities of race in the US:

Some time after English writer Henry Fairlie emigrated to the USA in the mid-1960s, he visited Thomas Jefferson’s Monticello plantation and took the standard tour. When the White guide asked for questions, Fairlie inquired, “Where did he keep his slaves?” Fairlie reports that the other tourists looked at him in disturbed silence, while the guide “swallowed hard” and said firmly that “the slaves’ quarters are not included in the official tour.” (Fairlie, 1985.) Housing segregation, and the systemic racism it reveals, are still not on the official tour.”

Two decades later, the question we must answer is whether we are willing, as scientists and citizens, to put housing segregation—and all the other institutions that do so much to dictate the vicissitudes of Black life—on the official tour of the USA.

## Systemic racial bias: the role of mental structures and resulting social interactions

We began with institutions and society. Now, we move to individual minds surrounded and shaped by these societal structures. Next, we then move to interacting minds, which further perpetuate societal and individual racial distinctions. Racial bias at each level supports bias at the other levels, creating a racist system.

To understand individual mental structures, we start with unconscious inference, identified by Helmholtz, and its heir, implicit bias, most relevantly as expressed by Whites associating Black racial cues with negative concepts. Socially motivated (mis)perception goes one stage earlier to bias information seeking and interpretation. More specific links among racial bias in perceiving physiognomy, linked to dehumanizing associations, and aggressive behavior close this first section on the individual.

### Unconscious inference

Among the intellectuals who contributed to the emergence of experimental psychology as an independent discipline in the nineteenth century was the German polymath, Herman von Helmholtz, whose numerous contributions to science include the concept of “*Unbewuste Schluss*” or “*unconscious inference*.” Helmholtz’s concept was simple, but its implications are profound, even more so today with recent advances in the mind and brain sciences. Given the complexity of just the visual world, how are humans to represent it based on their individual-level, meager sensory and perceptual system, which entails the shunting of packets of data from the world outside, through the eyes and into the brain? Helmholtz offered two ideas. First, perception is not veridical, given the complexity of the world and the rudimentary nature of the minds attempting to make sense of it. Second, as implied by the word *inference*, what one deduces from the evidence provided by the senses is not a replica of what is out there. Rather, mental representations of the physical world are mere approximations.

Whittling the self-esteem of Homo sapiens down further, Helmholtz went on to say that perception is not controllable, but rather that it unfolds automatically. He used a commonplace example to make this point. We know that it is not the Sun that rises, but rather that the Earth revolves around it. But when we sit on our porch at sunrise, and look toward the horizon, we incontrovertibly experience ourselves as being fixed, and the Sun, however bulky, pushing itself up to meet us. To say about the Sun that “it rises” is completely inaccurate yet completely compelling. That incorrect perception is not something over which we have choice. To think otherwise is to delude ourselves.

Helmholtz’s two ideas contained in the phrase “unconscious inference,” with many additional levels of social complexity, summarizes the challenge when we confront systemic racism. On the one hand, we “know” the facts about an economy purportedly mounted on free labor for 250 years, the undelivered promise of 40 acres and a mule, the failure of Reconstruction, the resistance to desegregation, the history of redlining and gerrymandering, a history of unequal access to education, jobs, housing, finance, healthcare, and a lack of equal protection under the law. On the other hand, the limited sensory, perceptual, learning, and memory systems of humans set up a built-in blindness and automatic inferences that generate the illusions that, for instance, White people experience more discrimination than Black people (Norton & Sommers, 2011). Or, if Black Americans have any challenges, they have created their own situation in America today (Pettigrew, 1979) and therefore are responsible for getting themselves out of that situation. Not that minorities have no illusions, but the illusions of the higher-status group have more consequences because they usually also have more power.

The features of human minds that feed into the production of systemic racism come in two forms: ordinary errors of perception, attention, learning, memory, and reasoning that are the hallmarks of all thinking systems with human-like intelligence. In addition, we add another level of theorizing familiar to psychologists, that of *motivated reasoning*, the idea that our preferences, goals, and desires can bias our reasoning and lead to prejudicial decisions and outcomes (Fiske & Taylor, 2021; Kunda, 1990).

Another hallmark of human cognition is the phenomenon of *loss aversion*, the finding human beings much prefer avoiding losses to acquiring equivalent gains (Kahneman & Tversky, 1979). Even as White Americans resist and deny the reality of systemic racism, they nonetheless feel the loss of White privilege and social status quite keenly, creating powerful resentments that motivate them to reason away the potential existence of systemic racism (Craig & Richeson, 2014; Parker, 2021).

### Implicit racial bias

Beginning in the 1980s, psychologists began to document a puzzling result. Individuals who claimed to have no racial animus showed evidence of negative attitudes and stereotypes toward Black Americans (Devine, 1989; Dovidio & Gaertner, 1986). Soon, the hunt for methods to better access “implicit bias” (as contrasted with standard, explicit bias measured in surveys) was underway, with specific calls for the invention of better technologies that could bypass conscious awareness or conscious control (Greenwald & Banaji, 1995).

One such measure, the Implicit Association Test (IAT), has demonstrated a wide array of group evaluative associations. Typically, people can pair own-group cues faster with positive concepts, and other-group cues faster with negative ones—compared with vice versa. For example, White and other non-Black Americans show robust race bias in their inability to associate “good” and “bad” equally rapidly with the social categories Black and White. The IAT has attracted considerable attention (see Greenwald et al., 2020, for best practices, reliable effects, and ongoing investigations). A public online location, since 1998, has provided data from millions of tests taken by volunteer participants at http://www.implicit.harvard.edu. Several signature results have replicated multiple times with large samples over time:

- Race bias is consistently visible in the data.
- A small positive correlation between stated and implicit race attitudes exists, but the two are largely dissociated, i.e., many of those who report being neutral (no negative explicit attitudes toward Black or White Americans), do carry implicit associations of Black + bad and White + good to a larger extent than White + bad and Black + good. This result prompts us to yet again note that the term “racism” has been used by contemporary psychologists to refer to conscious forms of race prejudice and to emphasize its semi-independence from less conscious or implicit forms of race bias. To make this distinction clear, researchers who study implicit race bias have gone to great lengths to reserve the term racism to only refer to conscious expressions of racial animus. Our usage of the term *systemic racism* in this article is undertaken is in the interest of including all levels of analysis (individual, institutional, societal) and all forms, from the most explicit to the most implicit. The result of a low correlation between explicit racism and implicit race bias makes the point empirically that the two are not the same. Of course, implicit race bias feeds into what may become racism, and for this reason it is best to think about implicit race bias as the roots of racism, not the above ground, visible structure. Implicit race bias also results from systemic racism.
- Asian Americans show the same pattern as White Americans, even though as a third-party group in response to a Black–White test, they might be assumed to have neutrality. From the point of view of systemic racism, this is an example of what it means to live in a system of inequity at all levels. Even third-party groups will acquire negative and positive attitudes toward groups that are not their own.
- Black Americans express strong positive feelings toward their own group but on the measure of implicit cognition, they show no preference for their own group, with scores of almost any sample of Black Americans showing relative neutrality, i.e., equal association of good and bad for Black and White Americans. This absence of ingroup-favoring attitudes—juxtaposed with the ingroup-favoring lack of neutrality in all other groups in the same society—is open to various interpretations, from moral balance to internalized racism to astute pragmatism; all await other data.
- Tests of anti-gay bias revealed it to be quite high in 2007 but steadily dropping off (by 64% since 2013) to be at an all-time low today. By comparison, anti-Black bias has dropped, but to a much lesser extent, by about 25% (Charlesworth & Banaji, in press). A 25% drop-off in race bias is not insignificant, and although the genders differ in magnitude of bias, both men and women are losing bias at equal speed. Although all demographic groups are changing, young Americans are changing faster than older Americans, suggesting that the world they inhabit is signaling a less biased set of attitudes.

Together, these data point to the individual manifestation of systemic racial bias, hidden from view but robustly present. However, psychologists have also gone beyond such demonstrations of basic cognitive associations as markers of implicit mental content to show that individual and institutional change is possible if the will to create change exists.

### Socially motivated (mis)perception

The idea of motivated reasoning or motivated cognition gathers several useful ideas to understand how individual humans shape and even distort perception to deal with real or perceived threats to self. Kunda (1990), for example, posited that the individual need for accuracy is thwarted by the demand to reach a conclusion prior to the evidence being satisfactorily in place and that one’s goals and motives often drive decisions. These decisions reveal many identifiable biases that emerge to weaken the orientation toward accuracy (see Fiske & Taylor, 2021).

With more direct focus on motivated reasoning as it concerns social change, Kay et al., (2009) presented empirical evidence for a motivated tendency to view things as they *are* and conclude that such a state of affairs exists because it is reasonable and even representative of how things ought to be. The connection to systemic racism is quite clear, as the authors further demonstrate that motivated cognition exists in the interest of justifying sociopolitical systems that maintain inequality and resist change. People justify the status quo, preferring stability especially if they are privileged, but even if not (Jost & Banaji, 1994). Groups in a secure position show the cultural equivalent of inertia, seeking stability, but groups on the move express inertia as continuing to move (e.g., acquiring mainstream standing) (Zárate et al., 2019).

Two substantive theoretical accounts undergird these ideas as they concern complex interactions of within-person and across-person phenomena such as systemic racism. First, Sidanius and Pratto’s (1999) Theory of Social Dominance offers evolutionary and cultural evidence to support the idea that hierarchies are an almost obligatory feature of human social groups. A related but independent idea may be found in Jost’s System Justification Theory (Jost, 2020), which explicitly makes the case that individuals will sacrifice self and group interest in order to maintain larger “systems” of social arrangements and work to keep them in place. The reason, Jost argues, is that such a motivation serves to meet deep psychological needs for certainty, security, and acceptance by others. The overarching social structure is important to protect because if it is stable, then all within it will be safe, including those disadvantaged by established hierarchies.

### Perception of phenotypes, deadly associations, and system-maintaining behavior

With regard to perceptions of race, the mere categorization of someone as Black shifts perceptions of their phenotype. For example, a series of experiments documented that people’s knowledge about race phenotypes drives perception of lightness of the skin tone (Levin & Banaji, 2006). In other words, experiments held skin-tone constant and varied only the features, from Afrocentric to Eurocentric; this variation in features shifts perception of skin tone, such that Afrocentric faces are viewed to be darker skinned than Eurocentric ones, despite the same gray-scale tone.

Skin tone and features are critical cues to make life and death decisions, especially in ambiguous situations that are often present in so many interactions between police and Black citizens. In simulations of police-citizen encounters, people are more likely to “shoot” unarmed Black men than otherwise equally unarmed White men (Correll, Wittenbrink, Park, Judd, & Goyle, 2010). Black men with more phenotypically Black features are more likely to receive the death penalty for murdering a White person, holding constant the features of the crime (Eberhardt, 2019). The phenotypicality effect extends even to Whites with Afrocentric features (Blair, Judd, & Chapleau, 2004). Judgments of criminality can be primed by a Black face (Eberhardt, 2019).

And there’s more: the race–crime association overlaps the dehumanizing association of Black faces with great ape faces, that Staples (2018) called the “racist trope that won’t die”; Goff, Eberhardt, Williams and Jackson (2008) provide evidence from policing that links apes and Black people, from the first moments of perception to the radio dispatch and other media, with systemic implications. In more recent work, Morehouse et al., (2021) have shown that White Americans associate White with *human* and Black, Asian, and Latinx with *animal* with greater ease than the opposite pairing (White with animal), regardless of the category of *animal* (generic or specific). Implicit racial biases (Whites favoring Whites) are consequential, correlating with judged trustworthiness and economic investment (Stanley, Sokol-Hessner, Banaji & Phelps, 2011).

More recently, Kurdi et al., (2021) measured attitudes toward a phenotypic feature that happens to be a dominant perceptual marker of race, Afrocentric and Eurocentric types of hair. First participants took an IAT measuring their implicit attitude toward Black women with natural or straightened hair. Then, subjects read a summary of a real legal case involving a corporation that fired a Black employee for refusing to change her natural hair (*Equal Employment Opportunity Commission* v. *Catastrophe Management Solutions*, 2016). The more negative the implicit attitude toward Afrocentric hair, the greater the sympathy with the corporation’s position rather than the plaintiff’s position in the legal case.

A relatively new approach to racial associations comes with the promise of epitomizing the term “systemic” in systemic racism. These are studies of large language corpora that are now possible using machine learning approaches to natural language. With the increasing availability of trained datasets—including large samples of the language of the Internet (content archives continuously collected by the nonprofit Common Crawl) or specific trained datasets of media such as books, TV shows, etc.—allow measuring the extent to which language contains attitudes and beliefs about Black and White Americans across time. Charlesworth and Banaji (in preparation) analyzed data from Google Books from 1800 to 1990. Setting aside the data from older books to focus on whether bias is present in the language today, these are the traits most associated with Black Americans (and not with White Americans) in the late twentieth century: *earthy, lonely, sensual, cruel, lifeless, deceitful, meek, rebellious, headstrong, lazy*. By contrast, these are the traits associated with White Americans (and not with Black Americans): *critical, decisive, hostile, friendly, polite, able, diplomatic, belligerent, understanding, confident*. Other work in natural language processing (NLP) sorts adjectives into 13 stereotype-content dictionaries (Nicolas, Bai, & Fiske, 2021). The above adjectives convey ambivalent reactions to Black Americans on several dimensions, but notably neglect competence; Whites in contrast feature several competence adjectives. NLP allows efficient analysis of language in the culture or in spontaneous, open-ended descriptions (Nicolas, Bai, & Fiske, under review).^4^

Words have an important role to play. People often express surprise about implicit biases in the minds of individuals who have no intent to harbor them. Considering how and why it occurs—plausible mechanisms—may prove convincing. One causal candidate is *language*, the predominant way humans communicate and express themselves. Words undertake much of the labor of creating racism in thoughts and feelings that are reflected in speech. Machine learning approaches to understanding racial bias in language will likely be a critical method to objectively uncover how words, spoken and written, create systemic racism. That is, linguistic patterns connect groups with valenced concepts, and the repeated pairings create associations. Without awareness, language produces the inbuilt in the architecture of social cognition (as an example, the NLP stereotype-dimensions dictionaries capture more than 80% of spontaneous stereotype content; Nicolas, Bai, & Fiske, under review).

### From cognitive racial bias to aggregate racialized behavior

Individual implicit attitudes have been repeatedly shown to predict behavior; Kurdi et al. (2019) offer the largest number of studies included in a meta-analysis to date. However, as the authors note, the actual attitude–behavior relationship is marred by the poor quality of many studies, especially given the lack of psychometric control over the predicted behavior. Among the controversies that have marked this work is an intriguing idea put forth by Payne, Vuletich and Lundberg (2017), who proposed that the small correlations between individual attitude and behavior must be acknowledged as a function of what they call the “bias of crowds,” the idea that an individual’s behavior is determined by the larger social context in which that individual exists. A number of studies have appeared recently to challenge the idea that individual attitude–behavior correlations is the right place to be looking. That the actual correlation between implicit attitude and behavior is larger than it may have appeared has been revealed in a series of studies that predict behavior at the aggregate level by using aggregate IAT scores by region, such as metropolitan areas, counties, and states. Charlesworth and Banaji (2021) reviewed these studies to demonstrate more substantive relationships between IAT racial bias and consequential social outcomes.

For example, the studies reviewed reveal that the greater the implicit bias against Blacks in a region (using average IAT scores of a region) the greater is the lethal use of force by police, the greater the Black American deaths from circulatory diseases, the lower is spending on Medicaid disability programs (more likely to assist Black Americans), the greater the Black–White gap in infant low birth weight and preterm births, the greater the Black–White gap in school disciplining (suspension, law enforcement referrals, expulsions, in-school arrests), the Black–White gap in standardized testing scores (3rd–8th grade for math and English), and lower upward mobility.

To grasp the meaning of systemic racism as it exists at the individual level within larger society, not just in a single moment by across time, a study by Payne, Vuletich and Brown-Iannuzzi (2019) is illustrative. Their analysis of IAT data today yields strong correlations with the ratio of enslaved to free people in the southern US in 1860. States with a larger ratio in 1860 are the states with greater race bias today, 160 years later (r = 0.64). This correlation is much larger in magnitude than even the correlation between regional IAT race bias and Black American representation across the US (r = 0.32). As Charlesworth and Banaji (2021) note, “the result also suggests that today’s Americans who live in regions with greater historical legacies of slavery must be acquiring the particles of race bias embedded in the social atmosphere. Systemic discrimination is a useful term in this case as it helps capture the pervasiveness of race bias as it extends across both space and time.”

*Summary.* As explicit bias decreased, measured forms of implicit bias have persisted, potentially attributable to racial segregation. White Americans have limited direct experience with Black Americans, so cultural associations substitute for more individuated impressions. Implicit associations of “Black-bad” and “White-good” are weakening, but far from neutral. Meanwhile, socially motivated (mis)perception favors these system-justifying biases. Together, they support a syndrome linking racial phenotypes, deadly associations, and system-maintaining behavior. Further, cognitive racial biases underpin aggregate racialized behavior. These are some cognitive-motivational mechanisms of systemic racism. Other mechanisms involve everyday interactions that perpetuate bias. In particular, predictable patterns of disrespect and distrust maintain the interpersonal racial divide.

### Racialized social interactions

Face-to-face behavior propagates bias. Individuals carry racial biases into their social settings largely by interacting with others. Repeated patterns of behavior that differ by race are, at a minimum, racialized (defined by race) and often experienced as racist. Individual racial biases, enacted in daily life, perpetuate bias, which then links the individual to the norms, scripts, and habits that constitute the social system. Interpersonal interaction conveys bias, intentionally or not. In scores of studies, White Americans distance themselves from Black interaction partners, express non-verbal discomfort, and avoid them (e.g., Dovidio, Kawakami & Gaertner, 2002; Richeson & Shelton, 2007; Word, Zanna & Cooper, 1974). In the aggregate, these patterns constitute the concrete manifestations of a racially biased social system.

We have already seen White people’s generically negative default associations with Black Americans, linking them to crime (untrustworthy) and to animals (incompetent). These reflect the two key stereotype dimensions in intergroup perception (Fiske, 2018): warmth and competence. These dimensions organize people’s perceptions of social systems: perceived competence reflects groups’ stereotypic status in society. The hierarchy supposedly reflects merit, so rank predicts their supposed competence and evokes respect—or supposed incompetence and disrespect. Besides groups’ status (competence), the other aspect of social structure is groups’ apparent cooperative or competitive goals, interdependencies that stereotypically predict warmth and trustworthiness. Cooperators on our side are nice; competitors are not. Stereotypes derive from social structural perceptions (status and interdependence), especially when people learn about others they might encounter (Fiske, Cuddy, Glick & Xu, 2002; Nicolas et al., 2021). Black Americans do not get a break on either dimension. And because these racialized perceptions derive from social structure, they pave the way for systemic racism. Consider the evidence for these two dimensions: competence and warmth in racialized perceptions and behavior.

### Disrespect communicates Whites’ view of Blacks as low status and incompetent

The default representation of Black Americans is low status (Dupree, Torrez, Obioha & Fiske, 2021). Whites spontaneously associate Black faces with low-status jobs, compared to Whites. The structural belief that Blacks are low status appears in associating them with jobs such as janitor, dishwasher, garbage collector, taxi driver, cashier, maid, prostitute. This race–status association correlates with endorsing social dominance (believing that some groups inevitably dominate others, and it is better that way) and with meritocracy (group get what they deserve). All these judgments share a common element of disrespect and assumed incompetence.

Race–status associations emerge in behavior that maintains Black people at the bottom of the hierarchy. Respondents endorsed Black applicants for lower status jobs and withheld support for organizations and government policies aiding minorities. Thus, racialized associations, assumptions, and preferences all identify a view of Black people's structural position as low status, on average. Behavior communicates these attitudes, whether examined or not. Thus, race–status associations imply Black incompetence, covarying with feeling-thermometer (0–100) ratings of interracial bias, social dominance orientation, meritocracy beliefs, as well as hierarchy-maintaining hiring and policy preferences.

Disrespectful behavior that presumes incompetence of Blacks appears in another series of studies. Well-meaning liberals, expected to introduce themselves to a Black partner, dumbed-down their speech, as they did in vocabulary for a task assignment (Dupree & Fiske, 2019). Similarly, White Democratic presidential candidates also showed a competence downshift in speeches to minority audiences only (Dupree & Fiske, 2019).

This pattern reproduces itself when respondents imagine introducing themselves to a lower-status person (race unspecified) at work (Swencionis & Fiske, 2016). They claim their goal is to communicate their own warmth (as they downplay their competence), but this rests on the presumption of the other’s incompetence. Trying to be folksy does not communicate respect.

The presumption that structural status predicts competence is widespread (averaging r > 0.80 across US and international samples; Fiske & Durante, 2016). The implication is that for most White Americans, the association that pops into their minds will link a Black person with incompetence. People communicate such disrespect by failing to bet on or invest in the other’s performance (Walsh, Vaida, & Fiske, under review).

Structurally, this amounts to racism. Black people are widely perceived as inferior in these ways, which are baked into the social hierarchy, reflecting disrespectful patterns of interpersonal behavior. All of this perpetuates the social hierarchy and the image of Blacks as incompetent.

Worse yet, disrespect surfaces in police encountering Black drivers. From the first moment (“Hey” instead of “Sir” or “Ma’am”), police officer language shows computationally derived, measurably lower respect (Voigt et al., 2017). Given the already fraught relationships between police and Black community members, this worsens an already dangerous encounter and undermines the chances to create trust.

### Distrust communicates Whites’ views of Blacks as uncooperative and not warm

Besides incompetence, the other major dimension of social cognition is warmth (trustworthy, friendly), as noted. The default stereotype of a Black person is probably also untrustworthy, but the data on this point are surprisingly indirect. Whites can be expected to distrust Blacks as part of the larger principle that, categorically, people mistrust outgroups. More specifically, as noted, Whites associate Blacks with crime, which certainly undermines trust.^5^ This configuration fits survey data showing that ratings of poor (i.e., explicitly low-status) Black people allege incompetence (disrespecting them) but also lack of warmth (distrusting them).

Plotting these ratings in a warmth x competence space, poor Blacks are frequently judged as low on both. Because White Americans link race and status, the low-income Black person is the default Black person, allegedly incompetent, but also untrustworthy. Mistrust is indicated by excessive surveillance of Black Americans (driving while Black, shopping while Black, false accusations of theft or assault, police shootings…).^6^

Distrust can be operationalized as behavior: In the economic Trust Game, a player must decide how much of their starting endowment to share, on the knowledge that it will be tripled, and on the hope that their partner will share back, generously. Incentivized trust-game behavior closely tracks warmth ratings; that is, societal groups rated as low warmth and untrustworthy receive less shared endowment, presumably because they are not trusted to share it back. In nationally representative samples, people of color do not fare well in the Trust Game (Walsh et al., under review). In more prosaic settings, non-verbal behavior reveals unmonitored dislike (if not specifically mistrust), as noted.

Black Americans experience repeated treatment as incompetent and untrustworthy. Because this stereotype and ensuing behavior is racially category-based and negative, as well as potentially controllable, it is racist. Because the behavior comes from societal stereotypes, which come from social structure,^7^ it is systemic.

### Whites’ potential control implies responsibility for reinforcing system racism

Racialized interactions could also be termed racist, in the sense that White people could potentially observe their own inequitable behavior if they chose (Fiske, 1989). People rarely examine these unwritten rules, typical behaviors, but conceivably they could, so “unexamined” bias captures the higher potential control for behavior than for implicit associations. Control implies responsibility in the minds of lay people and the law, so this interpretation of “racialized” as “racist” creates concern and is likely to be contested. But the science makes the empirical point here that racialized social behavior is demonstrably controllable, given sufficient incentive (Monteith, Lybarger & Woodcock, 2009; Sinclair, Lowery, Hardin & Colangelo, 2005). So systematically different behavior by race reflects a racist habit, script, or norm, the components of a system from the bottom up.

The challenge in controlling racist habits is that they are the cultural default. Much of this systematic behavior results from White Americans’ inexperience with Black Americans, thereby substituting societal representations for individuating information about the unique human (Fiske & Neuberg, 1990). People use especially those default representations that fit their natural human tendency to detect and prefer people they view as similar to themselves. To unpack this, consider some basic principles of affiliation that would predispose Whites to favor other Whites and exclude Black people. First is the basic tendency to categorize others and to favor those of the ingroup. For decades, principles of attraction have established its foundations in similarity (Byrne, 1971; Montoya & Horton, 2013) or homophily (McPherson, Smith-Lovin & Cook, 2001). And mere categorization suffices to produce ingroup favoritism (Tajfel & Turner, 1979). No animus is necessary, although it easily develops. As a basis for categorization, race is arbitrary (more so than gender and age; Kurzban, Tooby & Cosmides, 2001) but common (Sidanius & Pratto, 1999). Thus, race-based ingroup favoritism is a default, in the absence of other experience.^8^ This makes it hard to over-ride.

Societal segregation by race makes difficulties for overcoming the racial default. Segregation limits White exposure to Blacks, undermining their direct experience, leaving Whites to rely on cognitive shortcuts to represent Blacks as a group. Indeed, the less exposure people have to outgroups, the more clearly they differentiate among them–stereotypically. That is, White Americans who know the least about other races have the clearest stereotypes about them; the less diversity, the more differentiated their cognitive representations (Bai, Ramos & Fiske, 2020).

### What’s wrong with that?

As a scientific question, a skeptic might ask, what’s wrong with differentiating by stereotypes? One set of answers concerns the demeaning individual and face-to-face interaction, just addressed. The other answers pertain to sheer demographic diversity of Black Americans, covered next.

Given its racial history and ongoing systems, societal patterns and cultural stereotypes prevailing in the US tend to associate Blacks with low status and Whites with high status as noted. To the extent this race–status association has a kernel of statistical accuracy (Blacks are over-represented in low-status jobs), it fails several tests as an argument for using stereotypes as a constructive strategy of intergroup relations. First, it ignores variability, individuality, and (especially) Black diversity. Second, category-based thinking exaggerates perceived between-group variability and minimizes perceived within-group variability (Tajfel & Turner 1979; Taylor, Fiske, Etcoff & Ruderman, 1978). So “nouns that cut slices” (Allport’s, 1954 felicitous phrase for category labels) do violence to the human data. What’s more, society has civil rights laws protecting people from being judged by their group membership, so the consensus is that this is not only wrong, but illegal.

Race–status associations, in practice, ignore all the structural contributors to race–status associations, such as the neighborhood effects, already described. Whites assume meritocracy, believing that status accurately reflects individual competence (Fiske, Dupree, Nicolas & Swencionis, 2016); globally, the perceived status—perceived competence correlation hovers around 0.80. (The only countries where people are more cynical about the status-merit link are former Communist ones; Grigoryan et al., 2020.) The point here is that status has many antecedents, and not all of them are merit (or other personal, stereotypical explanations, e.g., innately good/bad at math). Systemic factors such as neighborhood, school, family resources, connections, and especially race all receive no mention in the meritocracy account.

Whites do differentiate Black Americans by subcategories, e.g., by status, specifically social class, viewing low-income Black people as incompetent and untrustworthy, but Black professionals as competent and trustworthy (Fiske, Cuddy, Glick & Xu, 2002). Black Americans themselves differentiate several subtypes of Blacks likewise along a social-class dimension (Fiske, Bergsieker, Russell & Williams, 2009).

Status-keeping shortcuts are easier to maintain without information to the contrary, such as experiencing human variability. Whites with less exposure to Blacks are more overtly prejudiced as a function of structural features such as rural residence, where they encounter less diversity (Bai et al., 2020), and lack of education, where they experience less variability of ideas. As a structural matter, segregated White rural residence also predicts lower school quality partly because of the American policy of locally funding schools; this creates an association between a weaker tax base, rural location, ethnic homogeneity, and overt bias. These systemic factors interact to produce prejudice. As an earlier section shows, the social structure permeates American arrangements since the arrival of Whites on native lands.

Nevertheless, for most Whites, their isolated lives make them inexperienced about their Black fellow citizens. Housing segregation disfavors most Whites in experience with diversity, making them often inept and naïve when speaking about issues that are facts of Black lives. This means that Whites rely on cultural shortcuts to understand the Black people whose life experience they do not know. These cognitive representations derive from perceived structural patterns such as race–status associations and race-resource unfairness (Krysan & Crowder, 2017).

We have seen that Whites’ racial beliefs are relatively automatic (implicit bias) and ambivalent (warmth/competence). The resulting associations (stereotypes) are more subtle than most people believe. They are consequently hard for anyone to detect in themselves (unexamined) or in any one person (under the radar), but the patterns appear systemically as aggregate biases. Supposing the aggregate biases are problematic, at least because they ignore variability, examine that more closely.

### Aggregate bias ignores diversity

So far, this review has described the relentless systems of racism that limit opportunity and outcomes by race. Many Black Americans nevertheless succeed despite the rigged system. Black diversity thus results from those who escape the system, but also from African and Caribbean immigration, and from intermarriage. For Black students enrolled at selective colleges, especially, the diversity of their backgrounds is the main fact that underscores their success (Charles, Kramer, Massey & Torres, 2021). Any given White student’s background is far more predictable than any given Black student’s, which potentially ranges from extreme disadvantage to extreme wealth. For that minority (a third) of Black students whose segregated neighborhoods entail underfunded schools, gang violence, and concentrated police violence, their presence in college testifies to extraordinary resilience (Charles, Fischer, Mooney & Massey, 2009).

Most non-Black people do not realize that Black Americans are more diverse than most American ethnic groups. Underestimating their variety allows an oversimplified image to dominate every level, from mind to society, making it a systemic racism. This section describes diversity based on place, intermarriage, immigrant experience, parent education, and sheer escape.

A century ago, most Black Americans lived in the rural South, but after the Great Migration, most lived in cities, often in the North, usually hyper-segregated, but with family roots in both the North and South. By the turn of the current century, Black American student bodies at selective colleges were the most diverse in history, more biracial, more immigrant, more middle or upper class, and equally identifying themselves as both American and as Black (Charles et al., 2021). Black students, even as elites, show “unprecedented variation in terms of racial origins, skin tone, nativity, generation, class, and segregation” (Charles et al., 2021, Ch. 10).

Clusters of characteristics and attitudes illustrate the variety. **Mixed-race students** identify less with being Black, are comfortable with both Blacks and Whites, see Whites as less discriminatory, and report deep parental involvement in their schooling and cultural experiences. Mixed race students also have more White friends and fewer Black friends than their monoracial peers and are more likely to date outside the group, especially with Whites. In addition, mixed-race students are less likely to join majority-Black organizations on campus, and thus report less intense interaction with Blacks*.* Psychologically, the White view of biracial individuals continues to demonstrate hypodescent, i.e., the view that biracial individuals belong to the less advantaged group, or the cognitive expression of the “one drop rule.” Combining the sociological and psychological angle demonstrates the lack of consistency between how biracial Americans are viewed and the way they see themselves.

Black students with an **immigrant background** are most comfortable with other Black students, and report having strict parents who expect obedience, respect, hard work, and family loyalty without hands-on, hovering involvement. First-generation immigrants, especially African immigrants (versus Caribbean ones), believe in meritocracy and see Whites as not so discriminatory. After a generation, idealism gives way to pragmatism: Hard work pays off. African immigrant origins predict reliably higher grades.

As for segregation, **Black students growing up with more exposure to Whites** feel closer to them but also view Whites as more discriminatory, a psychologically complex mental state to manage. In contrast, living in segregated neighborhoods especially exposes Black students to higher (the top third) levels of disorder and violence, leading them to view Whites as more distant and discriminatory. But parents are more protective, relying on strict discipline but not trying to use shame or guilt as an influence strategy (more frequent in Asian families).

As with all students, high-school GPA predicts college GPA. Besides that, again as with all students, Black women do better than Black men, as do those with **educated parents**. Differences in academic preparation vary by segregation in two ways: the more White students in their schools, the worse Black students’ grades but the higher their SATs, suggesting more rigorous standards. Thus, the portraits of Black college students are diverse; generalizations are unreliable, except perhaps for one: resilience in the face of systemic bias and a diversity of adaptations to a variety of challenges.

We document Black diversity here for these reasons: First, to avoid making the litany of systemic Black disadvantages the sole image conveyed here. Second, because of segregation, many White people, including University faculty, see a Black person on campus and—assuming they realize this is a student—they presume the person comes from a low-income background, unprepared for college, with uneducated parents, native born, but with little experience outside the imagined ghetto, etc. This may be true for some small fraction of students, but not just the Black ones, and not true of most Black students on campus today. A third reason to remind the reader of Black diversity on campus is to highlight experiences of inter-racial contact as important one mechanism for overcoming racial bias, and—if scaled up to integrated neighborhoods, schools, and workplaces—for shifting systemic racism.

### Contact: exposure to racial diversity

People with least exposure to diversity have the most differentiated images of the outgroups they have never met (Bai et al., 2020). And the prospect and first experience of diversity is not salutary; newly diverse contexts show lower well-being (Putnam, 2007; Ramos, Bennett, Massey & Hewstone, 2019). But over time, people get used to each other: well-being is higher and stereotypes melt into each, forming an undifferentiated cluster of people like us, mostly warm and competent.

Psychology has 70 years of research to explain how this works, following Allport’s (1954) contact hypothesis. In one meta-analytic perspective (Pettigrew & Tropp, 2006), intergroup contact reduces prejudice, the more it meets Allport’s conditions: shared goals, non-trivial interactions, authority sanctions, and rewarding results. Much of the process seems to be affect-driven. If the contact setting would afford the opportunity for friendship, the contact effect is stronger (Tropp & Pettigrew, 2005). This is a useful reminder that much prejudice is emotional, not cognitive. In fact, a meta-analysis of 50 years of research on racist attitudes found that they predict racist behavior the most when they are emotions (“hating them”) rather than stereotypes (“they are lazy”) or even simple evaluations (2 on a 5-point scale) (Talaska et al., 2008).

Nevertheless, the core element of successful contact, goal interdependence, does operate via information processing. In laboratory experiments, interdependence makes people attend specifically to unexpected, stereotype-inconsistent information, and they make dispositional inferences, generating an individualized coherent impression of the teammate (Ames & Fiske, 2013; Erber & Fiske, 1984). Neural signatures of mindreading prominently include the mPFC regions that reliably activate when people are inferring another’s predispositions. The mind-reading mPFC activates most for an interdependent partner’s stereotype-inconsistent attributes. Although supporting evidence includes these mechanisms, a subsequent meta-analysis (Paluck, Porat, Clark & Green, 2021) notes that few high-quality intergroup studies have focused on race per se, few look at adults, few are experiments. We have much to learn.

## Conclusion: systemic racism is individual/interpersonal and institutional/societal but rarely recognized

Segregated housing disadvantages many Black Americans, and its effects are far-reaching, not only in life opportunities and outcomes (education, employment, health, well-being) but also in the psychology of systemic racism. We have argued that case here. Most Whites fail to recognize and appreciate the growing diversity of America’s Black population, which has arisen from a mixture of Black resilience, a growing middle class, rising intermarriage, and global-South immigration. Generally, White Americans—because of the segregation perpetuated to sustain their advantage—have limited exposure to Black Americans, so their knowledge is indirect, and based on cultural caricatures. Segregation allows White people to be clueless about race, and because racial bias is more automatic, ambiguous, and ambivalent than people think, they fail to detect it in themselves and others. As a result, White people have many unexamined biases, undergirded by earlier stages of information processing (e.g., attention, perception, learning, memory, reasoning) that sustain such a lack of awareness. These cognitive errors and biases stem from lack of exposure, lack of the accurate evidence, and a lack of necessary knowledge.

The assumption here is that if people were simply made aware of the facts that have been described in the earlier sections, they would slap their palm to their head and immediately vote for reparations. But as readers may no doubt deduce on their own, confronting accurate data and internalizing it is not a smooth or pretty process. That our minds resist information that challenges certain types of prior beliefs is a fundamental discovery from the mind sciences. Basic cognitive processes such as motivated cognition help to maintain a lack of awareness of racial experiences as they exist on the ground. But no lack of awareness need exist.

The human ability for conscious awareness, deliberate thought, and the motivation to link values to behavior cannot be underestimated as vehicles of change. We have accomplished this regarding how we understand the relationship of Earth to our Sun, so we know it is not as it seems. If we choose, we can similarly put our minds to derive the best evidence to learn about the presence or absence of systemic racism. If we can acquire the appropriate knowledge (often hidden from our conscious perception), we will be more likely to remain open to evidence that shows its presence.

If we do not undertake this effort, it is at our own peril. If, in the twenty-first century, we cannot mount a new struggle to see the social world for what it is, we are by choice dooming ourselves to extended ignorance that will be costly to us, our society, and the world we inevitably leave to our descendants. Earlier we provided evidence about unexpected (by scientists) decreases in implicit sexuality bias (massive drop) and race bias (more modest change) since 2007. These data provide optimism that mental content that we cannot change at will is nonetheless capable of movement toward racial neutrality across the US.

In other words, who-we-have-been need not be the future-selves-we-are-becoming. Here, we demonstrated that grappling with the correct data is a necessary step on the path to understanding our role in the creation of systemic racism. Among the blind spots that we will need to shake off, once and for all, is the belief that racism is the product of a few bad people in our society, and that removing them from power will suffice to deal with the issue.

## Footnotes

1 Space and time preclude our covering the targets’ perspective, identity, resilience. Nor do we cover racial socialization in children.

2 Through the sensory and perceptual systems granted to our species by evolution, these dyadic and small-group social interactions evolve into larger and larger social units, such as the hundreds of so-called friends or millions of so-called followers on more recent forms of social media. Today we transcend ancestral, small-group interactions to generate larger-scale groups whose interactions occur on an exponential scale. The internet provides avenues for the high-speed transmission of individual attitudes, beliefs, values, as well as for propelling action across communities and nations. These communications have the potential to spread both social good and social harm, with explicit racial animus and implicit prejudicial bias being examples of the latter.

3 Using the most common measure of segregation (the dissimilarity index), in that year 94% Black metropolitan residents lived under conditions of “high” segregation (an index of 60 or greater on a 0–100 scale), meaning that at least 60% of Blacks would have to exchange neighborhoods with Whites to achieve an even distribution of the races across neighborhoods (Rugh & Massey, 2014). Moreover, in a subset of metropolitan areas, not only were Black residents unevenly distributed across neighborhoods, they were also isolated within overwhelmingly Black districts that were themselves densely clustered near the central business district, a geographic pattern that Massey and Denton (1989) labeled "hypersegregation.”

4 The NLP fits more traditional findings, a form of cross-validation. Based on content analysis of an 84-adjective checklist, the language describing Black Americans did not change much, across samples from 1933 to 2007 (Bergsieker, Leslie, Constantine, & Fiske, 2012, Study 4): The most recent data describe ambivalent view of sociality (aggressive, gregarious, passionate), and some specific stereotypes (loud, talkative, religious, loyal to family, sportsmanlike, musical, materialistic), but saying nothing about competence. Neglecting to mention an obvious dimension can reveal taboo topics, stereotyping by omission (Bergsieker et al., 2012).

5 Black people may distrust Whites, too, but they have less standing (status and power) to do damage.

6 An odd anomaly: Abundant research describes Black people’s generalized trust as lower then Whites’ generalized trust. Also, social science has studied Black Americans’ mistrust of government, business, healthcare, and education systems that have historically abused them (see next section). This would hardly seem puzzling enough to be the lion’s share of the trust literature and to eclipse White Americans’ pockets of mistrust. Specifically, no one seems to study Whites’ mistrust of Black people. Overlooking the obvious is one symptom of a systemic bias.

7 The combination of status-competence and warmth-trustworthiness creates remarkably stable perceptions of social structure (Durante et al., 2015). In social systems across the globe, middle classes are stereotypically competent and warm (trustworthy) whereas homeless people are neither. And in the mixed quadrants, rich people seem competent but cold, whereas old people seem well-intentioned but incompetent. These class and age patterns are nearly universal. In contrast, ethnic, racial, religious, and other cultural stereotypes are accidents of history, reflecting what subset of a group arrived under what circumstances. Compare stereotypes of Chinese railroad workers in the nineteenth century to stereotypes of Chinese entrepreneurs in the twenty-first century.

8 Implicit bias is difficult to monitor, as noted. Yet another way that prejudice goes undetected, is in its modern form, of being exhibited less as outgroup harm and instead as ingroup help (Greenwald & Pettigrew, 2014). Despite this ambiguity, the net effect is the same—just harder to detect, and even lauded, because helping is a prosocial act that garners praise.
